# A comparative study of mouse bone marrow mesenchymal stem cells isolated using three easy‐to‐perform approaches

**DOI:** 10.1002/2211-5463.13493

**Published:** 2022-10-17

**Authors:** Yalan Lu, Yunlin Han, Li Zhou, Guiying Shi, Lin Bai, Kewei Wang, Chuan Qin

**Affiliations:** ^1^ Beijing Engineering Research Center for Experimental Animal Models of Human Critical Diseases, Key Laboratory of Human Disease Comparative Medicine, National Health Commission of China, Institute of Laboratory Animal Science, Peking Union Medicine College Chinese Academy of Medical Sciences Beijing China

**Keywords:** bone digestion, density‐gradient centrifugation, mouse bone marrow mesenchymal stem cells, secretome, whole bone marrow‐adherent culture

## Abstract

Mouse bone marrow mesenchymal stem cells (mBM‐MSCs) are important for preclinical tissue regeneration and repair studies. In the present study, we isolated mBM‐MSCs using three easy‐to‐perform methods (whole bone marrow‐adherent culture, density‐gradient centrifugation, and bone digestion), and then compared the morphology, proliferation, differentiation, and paracrine factor profiles of the isolated mBM‐MSCs. Of these three isolation methods, the bone digestion method resulted in the highest quantity of mBM‐MSCs with high growth potential and moderate differentiation. Conversely, the mBM‐MSCs isolated through the whole bone marrow‐adherent method exhibited the lowest potency for proliferation and differentiation. The differentially expressed factors between mBM‐MSCs were primarily those involved in immune responses. The highly expressed secreted factors included cytokines/members of the chemokine family, growth factors, and protein binding/proteinase activity. These findings provide a fundamental reference for development of MSC isolation methods.

## Graphical abstract

Mouse BM‐MSCs were isolated using three easy‐to‐perform methods [whole bone marrow‐adherent culture (BM‐MSC‐A), density‐gradient centrifugation (BM‐MSC‐G), and bone digestion (BM‐MSC‐D)]. BM‐MSC‐D exhibited the highest quantity of mBM‐MSCs with high growth potential and moderate differentiation. The BM‐MSC‐A showed the lowest potency for proliferation and differentiation. The paracrine factors were mainly involved in chemokine family, growth factors, and protein binding/proteinase activity.

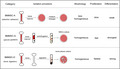

**Abbreviations**
BM‐MSC: bone marrow mesenchymal stem cell
CD29: integrin beta 1
CD34: CD34 molecule
CD45: protein tyrosine phosphatase receptor type C
CFU‐F: colony‐forming unit fibroblasts
FACS: fluorescence‐activated cell sorting
FBS: fetal bovine serum
GO: Gene Ontology
KEGG: Kyoto Encyclopedia of Genes and Genomes
mBM‐MSC: mouse bone marrow mesenchymal stem cell
MEM: minimum essential medium
MSC: mesenchymal stem cell
PBS: phosphate‐buffered saline
PS: penicillin–streptomycin
Sca I: suppressor of cancer cell invasion

Mesenchymal stem cells (MSCs) have the capacity to undergo localized self‐renewal and multilineage differentiation with low immunogenicity and tissue‐homing ability [1, 2, 3, 4]. They can communicate with *in‐situ* cells to modulate inflammatory processes, angiogenesis, and anti‐apoptosis [5, 6, 7]. These characteristics make MSCs an attractive candidate for tissue regenerative medicine [8, 9, 10]. MSCs have been isolated from different sources, including embryos, placenta, umbilical cord, adipose tissue, and bone marrow [11, 12]. Allogenic MSCs from embryos, placenta or umbilical cord may be not suitable for clinical application because of ethical issues and alloimmunogenicity. Autologous MSCs isolated from bone marrow or adipose tissue are more attractive options for therapeutic applications. Bone marrow mesenchymal stem cells (BM‐MSCs) were reported to exhibit a better therapeutic effect than those from adipose tissue, which can reduce the incidence of graft‐versus‐host disease [13, 14, 15]. Mouse models are popular and well‐studied tools for human diseases research and therefore mBM‐MSCs were chosen for subsequent research [16].

At present, the main approaches for isolating mBM‐MSCs include whole bone marrow‐adherent culture, density‐gradient centrifugation, and bone digestion [17, 18]. The whole bone marrow‐adherent culture relies on the adherent characteristics of MSCs in low serum growth conditions, periodically replaces non‐adherent cells, and achieves cell purification and expansion. To increase the yield of mBM‐MSCs, the density‐gradient centrifugation can be used to separate monocytes according to the specific gravity of mBM‐MSCs in the bone marrow. mBM‐MSCs are stromal cells in the marrow cavity and are an important part of the bone marrow in the hematopoietic microenvironment [19]. There are a large number of MSCs in the bone parenchyma. The bone digestion method isolates the mBM‐MSCs based on the migration and growth‐adherent abilities of mBM‐MSCs. In recent years, other methods have also been developed to isolate stem cells via flow cytometry and immunomagnetic beads according to membrane CD antigens [20]. However, stem cells derived from flow cytometry or magnetic beads are characterized by slow proliferation, high cost, and technical difficulties. These problems have limited the broad application of therapeutic stem cells.

The transplanted mBM‐MSCs migrate into damaged tissues and participate in tissue regeneration through various aspects of biological properties of mBM‐MSCs similar to other MSCs derived from other tissues. The evident therapeutic efficacy of MSCs appears to rely not only on the physical proximity of the transplanted cells within tissues, but also on paracrine cytokines released by MSCs, which are able to reduce cell injury and improve tissue repair capacity through both tissue regeneration and immunomodulation [12, 21].

The present study introduces three easy‐to‐perform methods, including whole bone marrow‐adherent culture, density‐gradient centrifugation, and bone digestion. The morphology, proliferation, molecular features, differentiation, and cytokine secretomes of mBM‐MSCs were analyzed and compared. The results of the present study may lay a strong foundation for stem cell isolation and preclinical applications.

## Materials and methods

### Cell isolation and culture

#### mBM‐MSC isolation

C57BL/6J mice (HFK Bioscience, Beijing, China) aged 6–8 weeks were killed via cervical dislocation and sterilized with 70% alcohol for 10–15 min. Femur and tibia were harvested using ophthalmic tweezers and scissors under aseptic conditions. Muscles and connective tissue around the bone were carefully removed as much as possible. Then, the clean bone was transferred to phosphate‐buffered saline (PBS) (#14190‐144; Gibco, Waltham, MA, USA) supplemented with 5% penicillin–streptomycin (PS) (#15070063; Gibco).

- mBM‐MSCs‐A were isolated from whole bone marrow‐adherent culture. Both ends of the femur and tibia were cut off, and bone marrow was flushed with PBS using a 1‐mL syringe. The marrow cavity was rinsed repeatedly until the flushing liquid was clear. Then, the tube with bone marrow was centrifuged at 400 g for 3 min. The supernatant was discarded, and the cell pellet was mixed well with complete medium and cultured in a 5% CO_2_ humidified incubator at 37 °C.
- mBM‐MSCs‐G were isolated using density‐gradient centrifugation. The bone marrow was obtained as the same procedure as that in the whole bone marrow‐adherent culture. The cell suspension was gently loaded onto the upper layer of the Percoll separation solution (#P8370; Solarbio, Beijing, China) with a density of 1.082 g·mL^−1^. The volume of cell suspension was twice that of the Percoll solution. After centrifugation at 260 g for 30 min, different layers were formed. An intermediate cloud‐like mononuclear cell layer was harvested and cultured in complete medium.
- mBM‐MSCs‐D were isolated from bone digestion. The bone was cut into 1–3‐mm^3^ pieces using scissors and tweezers. The bone pieces were washed twice in a new six‐well plate containing 5% PS. Next, they were transferred into a 15‐mL centrifuge tube containing 2 mL of 100 μg·mL^−1^ collagenase II (C6885; Invitrogen, Waltham, MA, USA) solution. The bones were digested at 37 °C for 1 h with frequent shaking. Then, 3 mL of medium was added to neutralize the digestion solution, followed by a centrifugation at 400 g for 5 min. The supernatant was then discarded, and the bone fragments were washed once again and cultured in α‐minimum essential medium (MEM) medium containing 10% fetal bovine serum (FBS) and 1% PS in a six‐well plate. When the cells were passaged, the residual bone fragments were put in a new culture dish for additional primary culture of mBM‐MSCs.

#### mBM‐MSC culture

mBM‐MSCs were cultured in α‐MEM (#12571‐063; Gibco) supplemented with 10% FBS (#10099141C; Gibco), 100 U·mL^−1^ penicillin, and 100 μg·mL^−1^ streptomycin (#15070063; Gibco). Cells were grown in a 5% CO_2_ humidified incubator at 37 °C for 24–48 h, followed by a replacement of half of the medium. When the cells reached 70–80% confluence, the supernatant was discarded and the cells were washed twice with PBS. The cells were then digested with 0.25% trypsin (#25200‐056; Gibco) and harvested. The cell passage of mBM‐MSCs was in a ratio of 1 : 3. The culture medium was replaced every 2–3 days. The cells in passage 2 and 3 were frozen and used for the subsequent experiments.

### Proliferation and colony‐forming unit fibroblasts (CFU‐F) assay

The proliferation of mBM‐MSCs was tested using a CCK‐8 assay. The third generation cells were seeded at a density of 2 × 10^3^ cells per well in 96‐well culture plates. After 1, 2, 3, and 4 days, 100 μL of 10% CCK‐8 solution was added to each well, followed by incubation at 37 °C for 1 h. Absorbance was measured at 450 nm using a microplate spectrophotometer. For the CFU‐F assay, cells were plated in six‐well plates (2000 cells of passage 3 per well) and allowed to incubate for 2 weeks. The cells were then fixed by methanol, followed by staining with crystal violet for 10 min, and the colonies were then counted manually.

### Flow cytometry analysis

The cells were labeled with markers in accordance with manufacturer's instructions. Briefly, the third generation of cultured mBM‐MSCs was harvested and centrifuged at 400 g for 5 min. Then, 2 × 10^6^ cells were re‐suspended in 100 μL of PBS and incubated with a series of antibodies (anti‐CD29 antibody: #12‐0291‐83; anti‐suppressor of cancer cell invasion (Sca I) antibody: #25‐5981‐82; anti‐CD34 antibody: #11‐0341‐81; and anti‐CD45 antibody: #45‐0451‐82; Invitrogen) at 4 °C for 30 min. The cells were then washed with PBS, filtered through a 70‐μm nylon mesh, and detected using flow cytometry.

### Cell differentiation

- Osteogenetic differentiation. The third generation of mBM‐MSCs was seeded in six‐well plates coated with 0.1% gelatin at a density of 1.92 × 10^5^ cells per well. When the cell confluence reached 70%, 2 mL of osteogenic differentiation medium was added to each well. The medium was replaced with fresh medium every 3 days. After induction of differentiation for 21 days, cells were fixed with 4% formaldehyde and stained with an Alizarin Red S solution (MUBMX‐90021; Cyagen, Santa Clara, CA, USA) for 5 min. The percentage of Alizarin Red S‐positive areas of the whole fields was measured and quantified using imagej (NIH, Bethesda, MD, USA) In total, five fields of mineralization levels per group are shown as a histogram.
- Adipogenic differentiation. The third generation of mBM‐MSCs was seeded in six‐well plates at a density of 1.92 × 10^5^ cells per well, and cultured with α‐MEM complete medium. When the cell confluence reached 100% or over‐fusion, the adipogenic differentiation medium was added. The cells were first cultured with liquid A (containing glutamine, insulin, IBMX, rosiglitazone, and dexamethasone) for 3 days, and then the culture medium was replaced with liquid B (containing glutamine and insulin) and cultured for 24 h. The B solution was then replaced with A solution. These two induction media were exchanged several times until lipid droplets were observed, followed by Oil Red O staining (MUBMX‐90031; Cyagen). The number of Oil Red O positive staining per field was counted using imagej. In total, six to nine fields of lipid droplet numbers per group are shown as a histogram.
- Chondrogenic differentiation. The third generation of mBM‐MSCs (3–4 × 10^5^ cells) was transferred into a 15‐mL tube and centrifuged. The supernatant was discarded and the premix (containing dexamethasone, ascorbic acid, insulin‐transferrin‐selenium, sodium pyruvate, and proline) was added, followed by another centrifugation. After one wash, the cell pellet was re‐suspended with 0.5 mL of complete chondrogenesis‐induction medium (containing dexamethasone, ascorbic acid, insulin‐transferrin‐selenium, sodium pyruvate, proline, and transforming growth factor‐β3), and cultured in a 5% CO_2_ humidified incubator at 37 °C. The chondrogenesis‐induction medium was replaced with fresh medium every 2 days. After chondrogenesis induction for 21–28 days, cartilage spheres were fixed, paraffin‐embedded, sectioned, and stained with Alcian blue (MUBMX‐90042; Cyagen). The percentage of Alcian blue positive areas of the tissue fields was measured and quantified using imagej. In total, five fields of mineralization levels per group are shown as histogram.

### Antibody array analysis

The antibody array analysis was performed using a mouse cytokine array Q400 (RayBiotech Inc., Norcross, GA, USA) in accordance with the manufacturer's instructions. The assay is a multiplex ELISA‐based quantitative array platform that can detect 200 mouse cytokines. Briefly, the mBM‐MSCs obtained from three isolation methods were cultured until they reached 80% confluence, and then the culture cells were washed with PBS and incubated with serum‐free α‐MEM. After 24 h, the conditioned medium was collected and concentrated using an Amicon Ultra (UFC901096; Millipore, Burlington, MA, USA) before analysis. The array membranes were incubated in a blocking buffer for 30 min, and then incubated with a biotin‐conjugated antibody. After three washes, the membranes were incubated with fluorescent dye‐conjugated streptavidin for 1 h, and the signal intensities were quantified with an Array‐Pro Analyzer^®^, version 4.5 (Mediacy Bernetics, Inc., Bethesda, MD, USA). Measurements of cytokines and concentration, as well as differential gene expression and pathways analyses, were conducted by RayBiotech, Inc., (Guangzhou, China). *P*_adjusted_ < 0.05 and fold‐change > 1.2 or < 0.83 were considered statistically significant.

### Ethical approval

All protocols in the present study involving mice were reviewed and approved by the Institute Animal Care and Use Committee at the Institute of Laboratory Animal Science, Peking Union Medical College (WKW19001).

### Statistical analysis

All experiments were repeated at least three times. The difference among the three groups was analyzed by one‐way ANOVA followed by Dunnett's test. Statistical analyses were performed using the prism, version 8.0 Software (GraphPad Software Inc., San Diego, CA, USA). All data are presented as the mean ± SEM. *P* < 0.05 was considered significantly different.

## Results

### Morphological characteristics of mBM‐MSCs from three isolation procedures

**Fig. 1:**
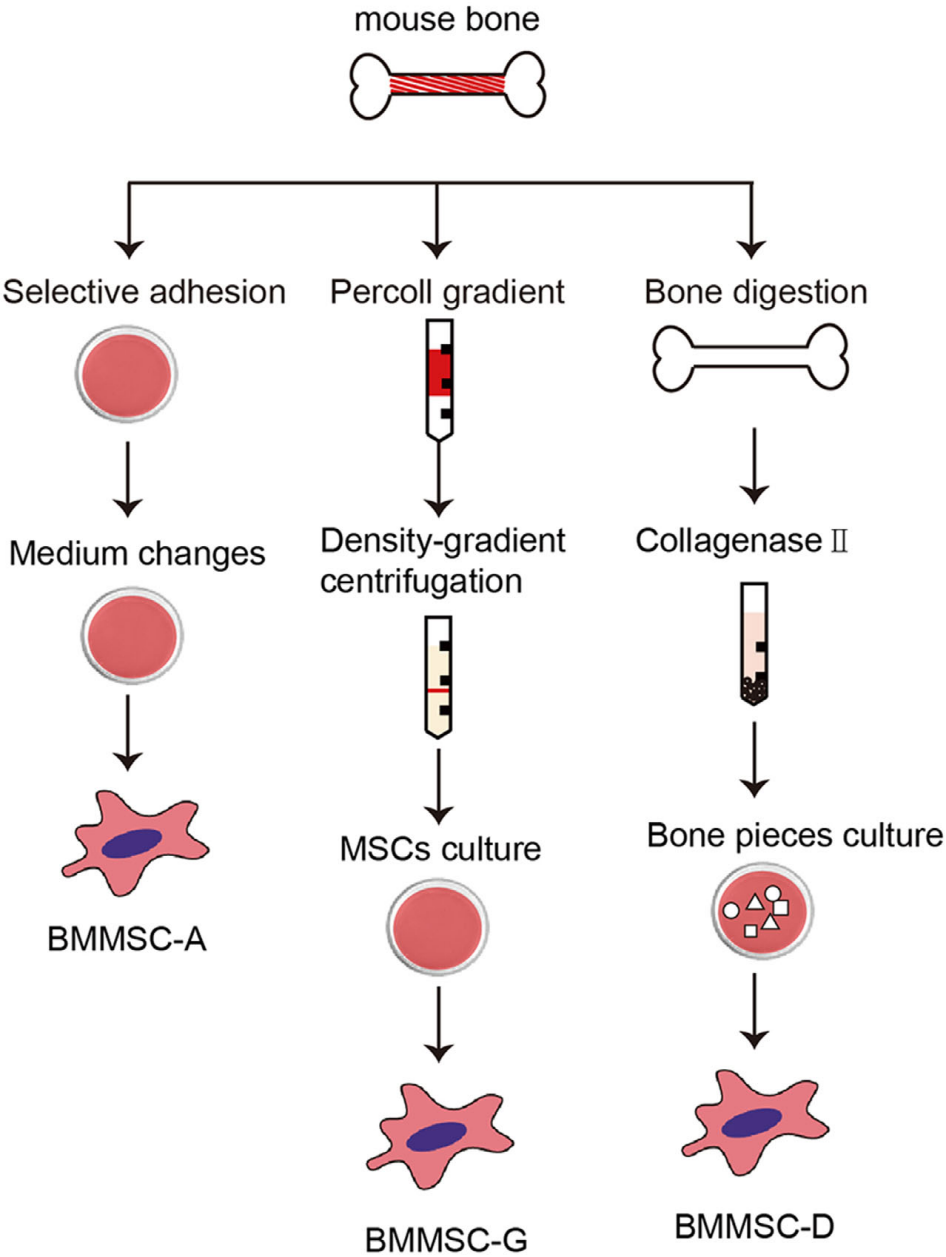
Flow chart of isolation of mBM‐MSCs using various isolation methods; BM‐MSC‐A, mBM‐MSCs from whole bone marrow adherent culture; BM‐MSC‐G, mBM‐MSCs from density‐gradient centrifugation; BM‐MSC‐D, mBM‐MSCs from bone digestion method.

**Fig. 2:**
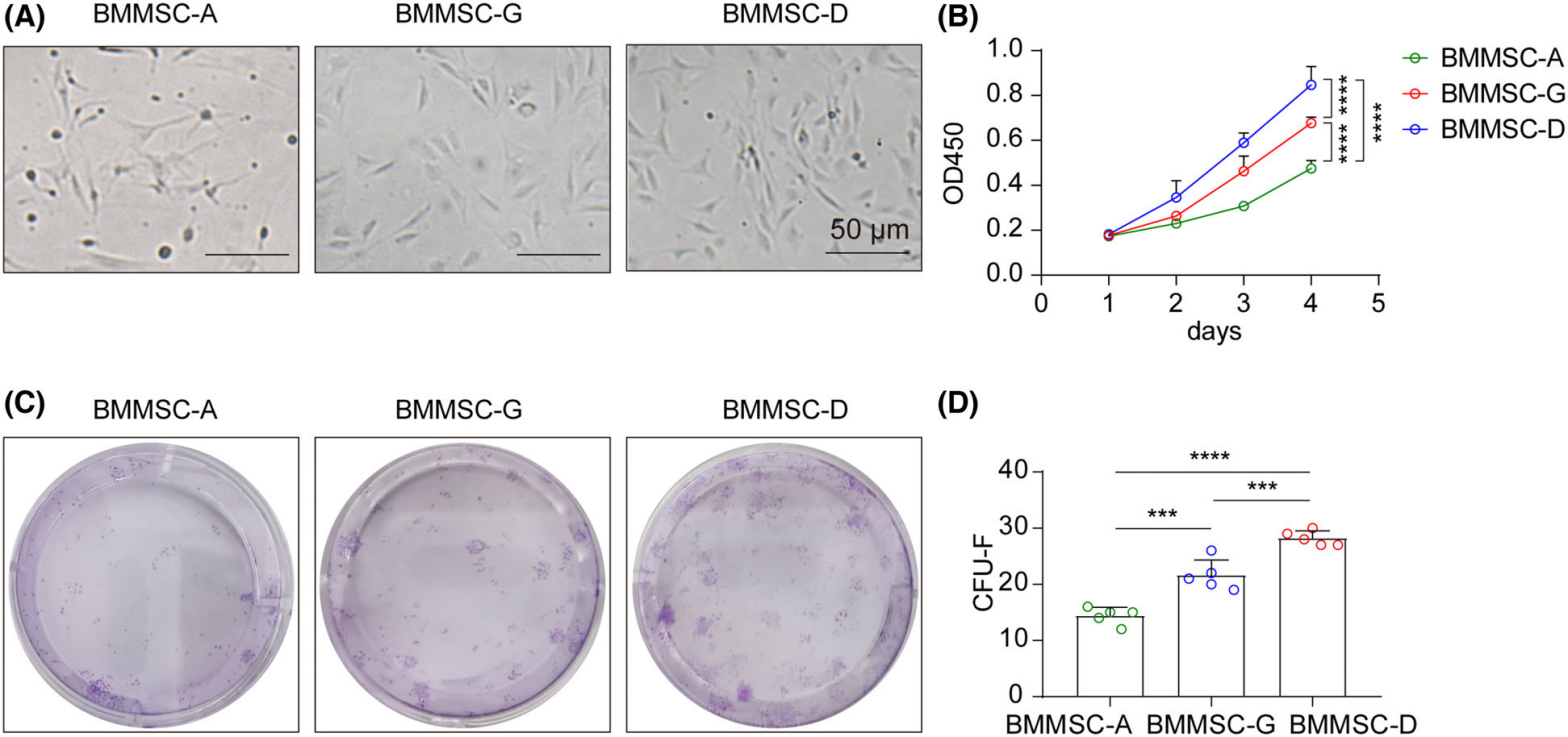
Morphology and proliferation of mBM‐MSCs obtained using three isolation approaches. (A) The morphology was viewed using an inverted microscope; scale bar = 50 μm. (B) Growth curves were measured by a CCK‐8 assay. (C, D) Colonies were stained with crystal violet (C) and CFU‐F was counted (D) (*n* = 3). Data are analyzed by one‐way ANOVA and expressed as the mean ± SEM, ***P < 0.001 and ****P < 0.0001.

To identify the optimal method for isolation of mBM‐MSCs, three easy‐to‐perform isolation methods are summarized in a flow chart (Fig. 1). In primary culture, mBM‐MSCs were mixed with several hematopoietic cells, but they did not survive for a long time. At the passage stage, all mBM‐MSCs were presented with a similar spindle shape. In the generation passage, the mBM‐MSCs showed homogeneous features when isolated via bone digestion (mBM‐MSCs‐D) and density‐gradient centrifugation (mBM‐MSCs‐G). The mBM‐MSCs in the whole bone marrow‐adherent culture (mBM‐MSCs‐A) grew some cells with an irregular shape (Fig. 2A). We then analyzed their growth properties by CCK‐8 (4 days) and CFU‐F (14 days) assays. mBM‐MSCs‐D appeared to grow faster than mBM‐MSCs‐G and mBM‐MSCs‐A, and mBM‐MSCs‐A showed the slowest growth (Fig. 2B–D). Moreover, the digested bone splices could be re‐cultured two or thee times, yielding more mBM‐MSCs. The results showed that bone digestion method can obtain more MSCs, which showed faster proliferative potential.

### Fluorescence‐activated cell sorting (FACS) detection of surface antigen molecules of mBM‐MSCs

**Fig. 3:**
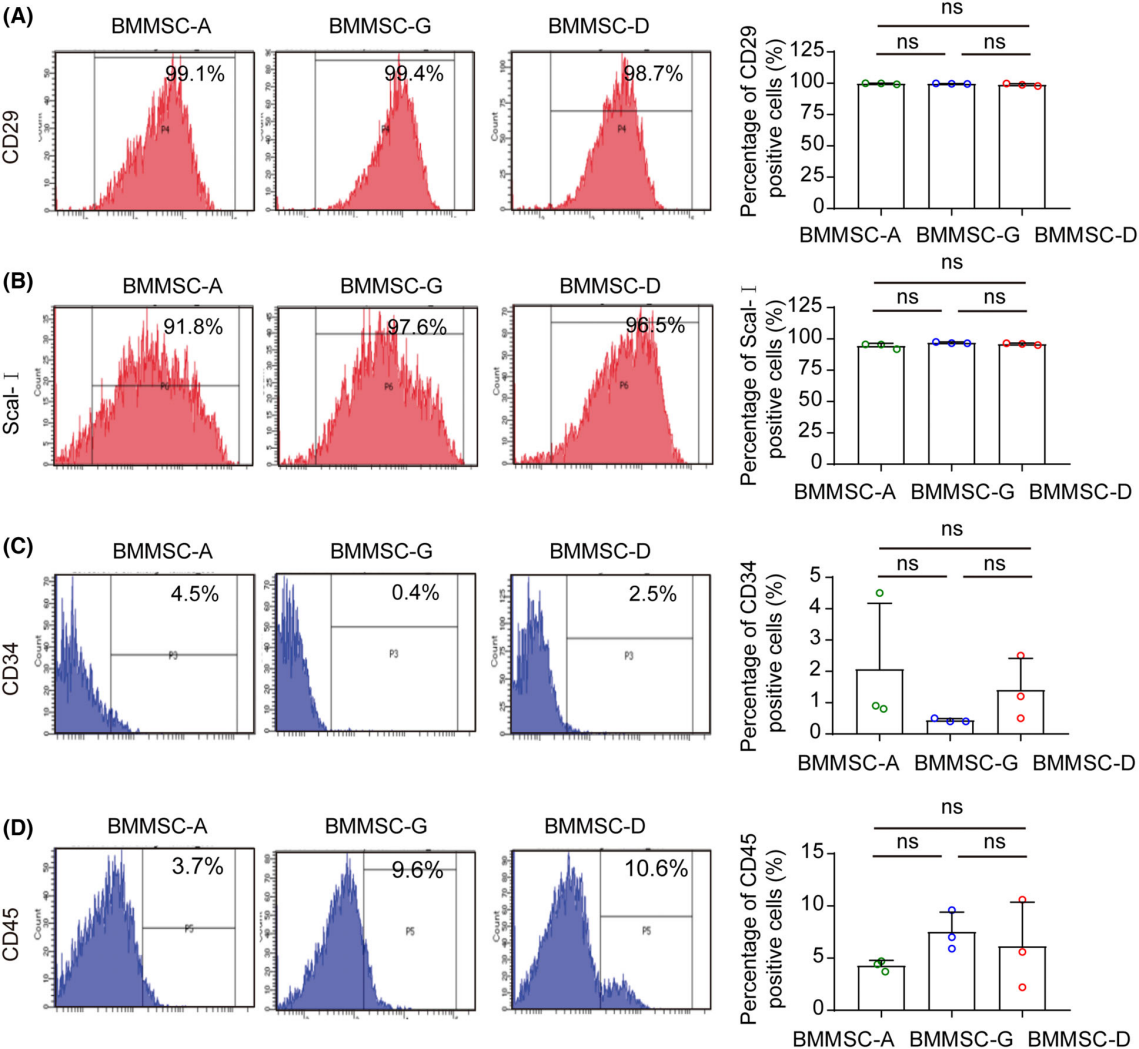
Cell markers of MSCs were analyzed by flow cytometry. (A–D) Surface antigen markers CD29 (A), Sca I (B), CD34 (C), and CD45 (D) were detected and quantified from the three groups (*n* = 3). Red markers represent positive surface antigens; blue markers represent negative surface antigens. Data are expressed as the mean ± SEM; ns, not significant.

The surface antigens of the mBM‐MSCs at passage 3 were further analyzed using FACS. The CD29 and Sca I are the positive markers, whereas CD34 and CD45 are negative markers of MSCs. The FACS results indicated that the positive rates of CD29 and Sca I were over 90%, whereas the positive rate of CD34 was less than 5%, and the positive rate of CD45 on the mBM‐MSCs was less than 15% from all the three isolation procedures. There were no significant differences in CD29, Sca I, and CD34 and CD45 markers between the three types of MSCs (Fig. 3). All of the mBM‐MSCs expression profiles of molecular markers were consistent with the surface antigen characteristics of MSCs.

### Differentiation potency of mBM‐MSCs into osteocytes, adipocytes, and chondrocytes

**Fig. 4:**
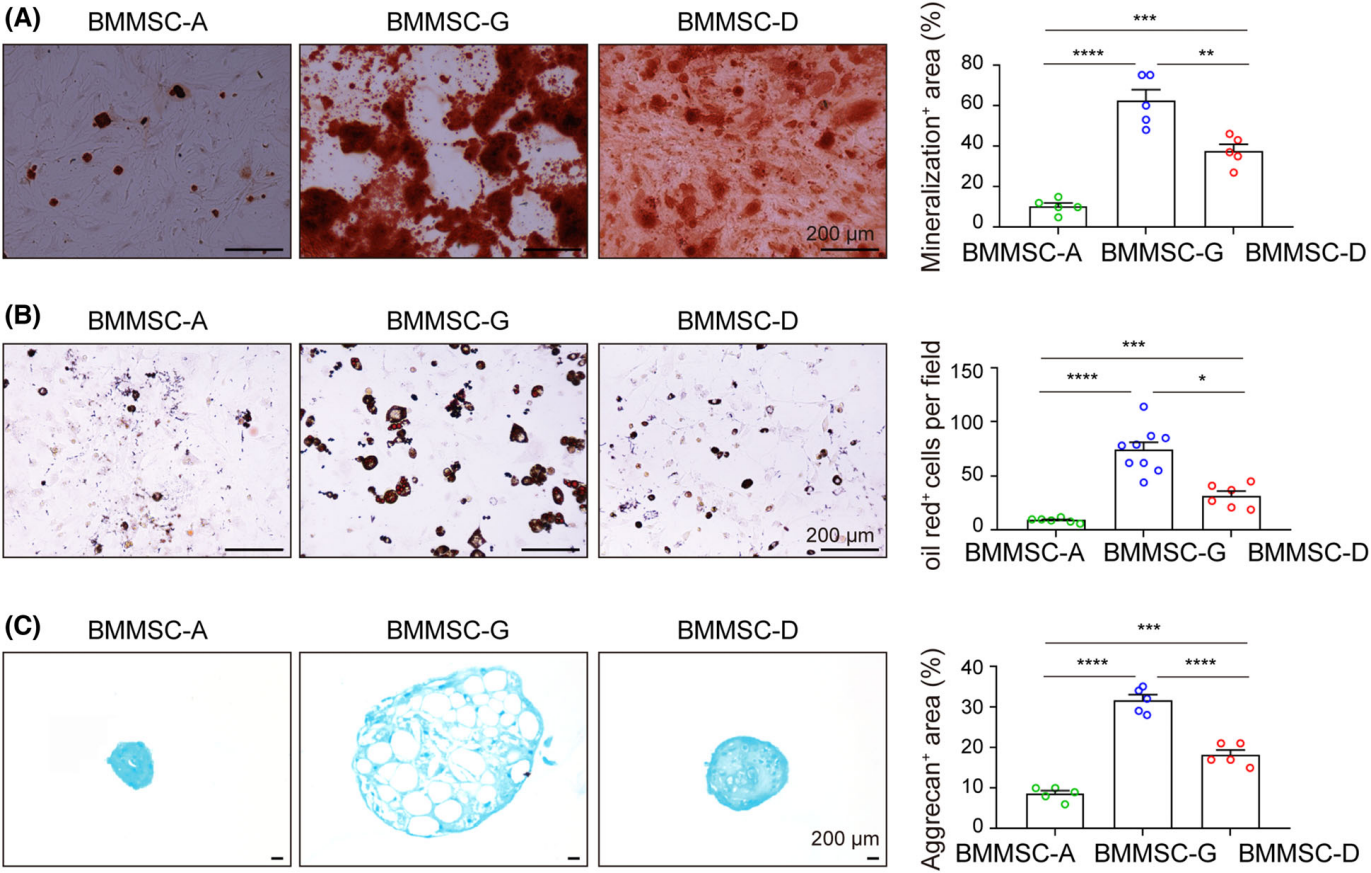
Differentiation potency of mBM‐MSCs. (A) Representative alizarin red S staining following osteogenetic induction medium culture. The histogram (right) shows the quantification of mineralization levels (*n* = 5). (B) Representative oil red O staining and quantification of adipocyte number per field for each of the three mBM‐MSC groups (*n* = 6–9). (C) Representative Alcian blue staining and quantification of chondrogenic differentiation from three isolations of mBM‐MSCs (*n* = 5). Scale bar = 200 μm; data are analyzed by one‐way ANOVA and expressed as the mean ± SEM, *P < 0.05, **P < 0.01, ***P < 0.001, and ****P < 0.0001.

The differentiation potency of mBM‐MSCs isolated from different approaches was compared at passage 3. When cultured in osteogenic medium, the mBM‐MSCs‐G displayed more calcium nodules than mBM‐MSCs‐D as analyzed by Alizarin Red S staining. There were fewest Alizarin Red S positive cells from the mBM‐MSCs‐A among the three methods (Fig. 4A). The adipogenic and chondrogenic differentiation results are consistent with osteogenesis. All experimental groups of mBM‐MSCs displayed lipid droplets or Alcian blue positive staining. mBM‐MSCs‐G did not display lipid droplets until day 7 and those of the other two methods did not until day 12. The mBM‐MSCs‐G displayed about three times more Oil Red O‐positive cells compared to those derived from the bone digestion method. There were relatively fewer lipid droplets in mBM‐MSCs‐A compared to the other two methods (Fig. 4B). The aggrecan positive area was successively decreased in mBM‐MSCs derived from the density‐gradient centrifugation, bone digestion, and whole bone marrow‐adherent culture, respectively (Fig. 4C and Table S1).

Overall, the mBM‐MSCs derived from density‐gradient centrifugation demonstrated the strongest differentiation capacity, with the bone digestion method displaying less and the whole bone marrow‐adherent culture demonstrating the weakest differentiation potential.

### Identification of secretome released by mBM‐MSCs

**Fig. 5:**
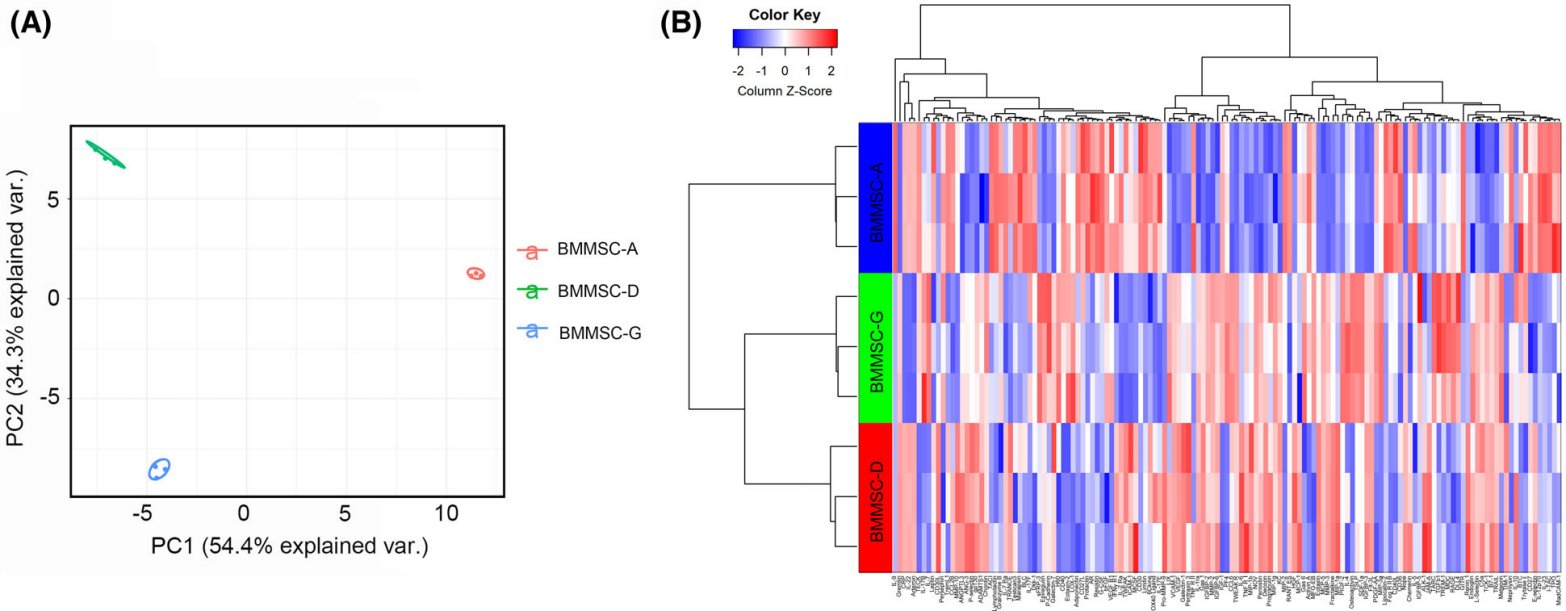
Secretome released by mBM‐MSCs. (A) Principal component analysis of expression profiles of mBM‐MSCs from three experimental groups. PC1 and PC2 refer to two different principal components. (B) In total, 139 significantly differentially expressed genes were selected and hierarchically clustered in a heatmap.

The secretive factors from the conditioned medium were then analyzed using antibody arrays, which involved growth factors, angiogenesis, cytokines, chemokines, cell‐adhesion molecules, and protein tyrosine kinase (Table S2). Principal component analysis showed that factors from mBM‐MSCs‐G exhibited a higher similarity with mBM‐MSCs‐D than mBM‐MSCs‐A (Fig. 5A). The cluster abundance from the heatmap confirmed this conclusion. The 139 differentially expressed proteins are listed in Fig. 5B. There were 47 and 55 cytokines that were markedly increased or decreased in the media of mBM‐MSCs from whole bone marrow‐adherent culture compared with the other two groups, respectively (Fig. 5B).

### Functional analysis of the differentially expressed secretive factors

**Fig. 6:**
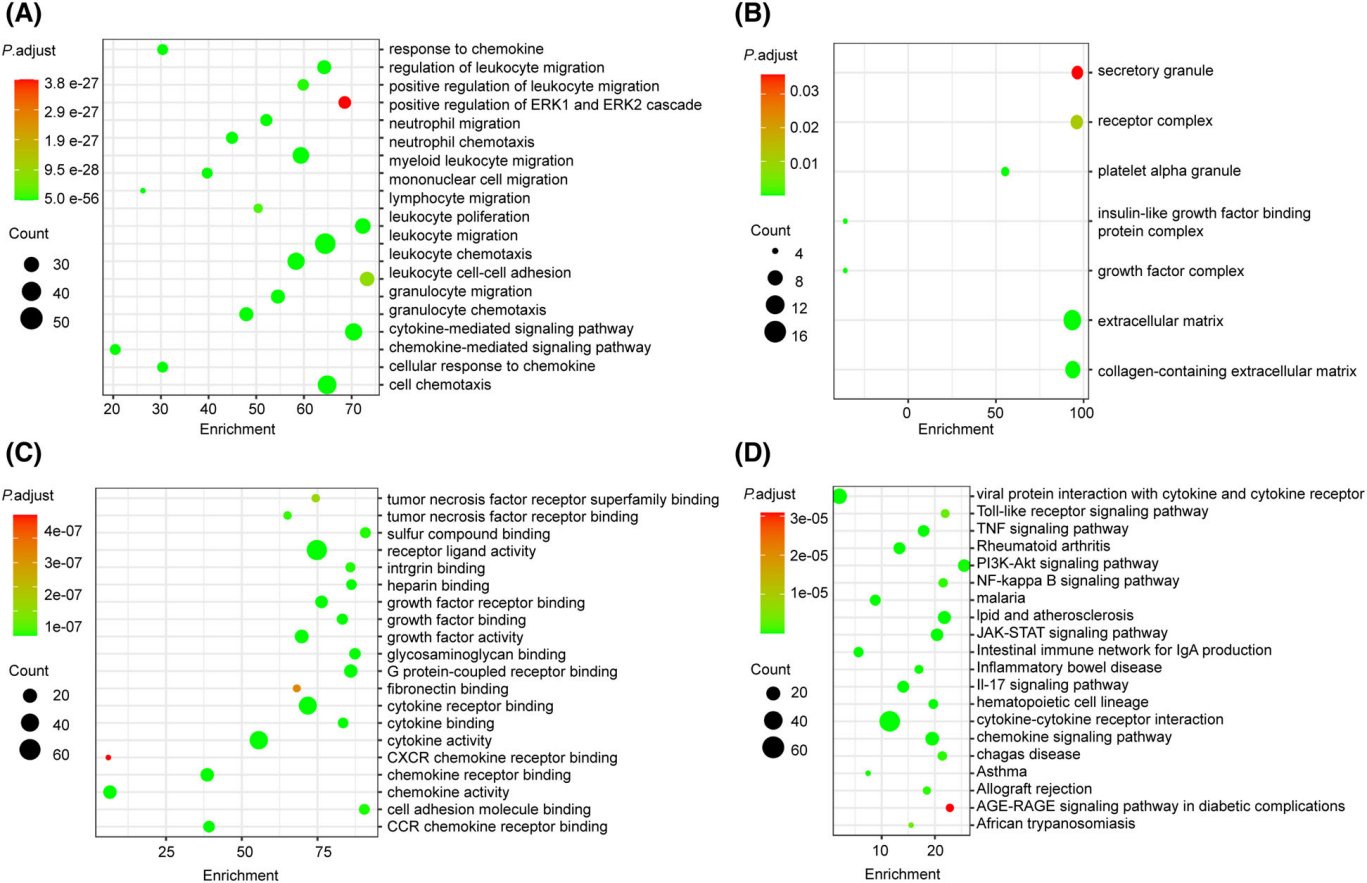
Functional analysis of differentially expressed mBM‐MSC secretive factors. (A–C) GO terms [biological progress (A), cellular component (B), and molecular function (C)] enrichment analysis among the three experimental groups were measured using david. (D) Bubble diagram of significantly enriched KEGG signaling pathways of the differentially expressed mBM‐MSC secretive factors.

Gene Ontology (GO; http://geneontology.org) analysis indicated that these differentially expressed paracrine factors were primarily attributed to immune response biological processes, such as response to chemokine/cytokines, and immune cell migration/chemotaxis (Fig. 6A). The cellular components were highly involved in secretory granule, receptor complex, platelet alpha granule, insulin‐like growth factor binding protein complex, growth factor complex, and extracellular matrix (Fig. 6B). The molecular factors were mainly involved in inflammation and growth binding/activity (Fig. 6C), such as tumor necrosis factor superfamily/sulfur compound/integrin/heparin/cytokine/chemokine binding, and growth factor binding. Consistent with GO analysis, the the Kyoto Encyclopedia of Genes and Genomes (KEGG; https://www.genome.jp/kegg) pathways enriched were mainly lying on immune pathways, such as Toll‐like receptor signaling, nuclear factor‐kappa B signaling, Janus kinase‐signal transducer and activator of transcription signaling, phosphatidylinositol‐3‐kinase‐Akt signaling, and the interleukin‐17 signaling pathway (Fig. 6D).

### Quantification of highly expressed cytokines of mBM‐MSCs

**Fig. 7:**
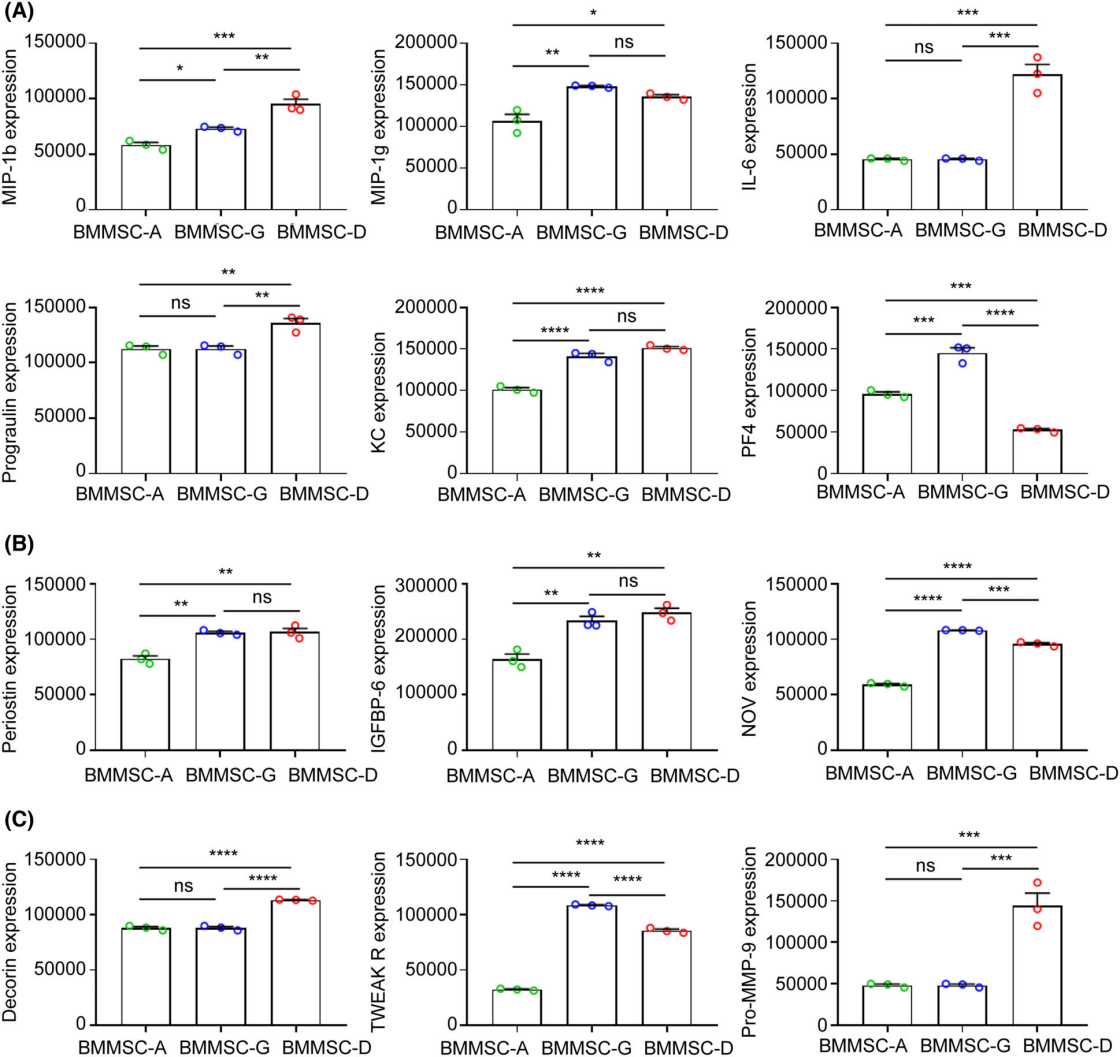
Quantification of highly expressed cytokines of mBM‐MSCs. (A–C) Histogram representing the highly expressed cytokines/chemokines (A), growth factors (B), and protein and proteinase (C) (*n* = 3). Data are expressed as the mean ± SEM; ns, not significant; *P < 0.05, **P < 0.01, ***P < 0.001, and ****P < 0.0001.

In total, 12 factors exhibited the strongest fluorescence signal intensity with values greater than 100 000, which were implicated in mBM‐MSCs biology (Fig. 7). MIP‐1b, MIP‐1g, interleukin‐6, progranulin, KC, and PF4 are involved in the cytokines/chemokine family. Periostin, IGFBP‐6, and NOV are growth factors. Decorin, TWEAK R, and Pro‐MMP‐9 are important regulators in protein binding and proteinase activity. The paracrine factors of mBM‐MSCs derived from the bone digestion method demonstrated the highest expression, whereas those from the whole bone marrow‐adherent culture exhibited the least expression, except for a decrease of PF4 and TWEAK R in the bone digestion group (Fig. 7).

## Discussion

mBM‐MSCs comprise a population of mesenchymal stromal cells that have been characterized as non‐hematopoietic skeletal progenitor cells playing a key role in the hematopoietic microenvironment [17]. As a result of their self‐renewal ability, multilineage differentiation, and low immunogenicity characteristics, autologous mBM‐MSCs present numerous advantages for use in tissue regeneration and injury repair. mBM‐MSCs are a critical preclinical source for investigating stem cell therapy and regenerative medicine, especially in the context of refractory diseases.

In the present study, three methods, including whole bone marrow‐adherent culture, density‐gradient centrifugation, and bone digestion, were performed to isolate mBM‐MSCs. When comparing the three approaches, the whole bone marrow‐adherent culture was an economic and easy method that did not require special reagents, although it was the weakest method for efficiency and differentiation. This approach is relatively non‐specific despite the use of selected sera and media for culture initiation, and the initial contaminating cells present may impact the proliferation of MSCs [22]. Moreover, this method may leave behind potential progenitor cells residing at the endosteum [23]. The density‐gradient centrifugation approach was a good choice for the separation of mBM‐MSCs with high purity and strong differentiation potency. However, this method did not capture all of the MSCs released by flushing the bone cavity because some cells were lost during the centrifugation step. This method therefore requires more mice for the isolation and culture of mBM‐MSCs. The bone digestion approach was an optimization for most experiments. The separation of mBM‐MSCs is a result of their high yield and strong proliferative capability, and is easy‐to‐perform. However, because the mBM‐MSCs derived from bone digestion need more time to migration to medium, they cannot be immediately used for primary transplantation, whereas mBM‐MSCs derived from whole bone marrow and density‐gradient centrifugation can be transplanted for therapy directly (Table S1).

mBM‐MSCs can be differentiated into target cells through their tissue‐homing ability to repair the damaged tissues. Autocrine and paracrine cytokines derived from mBM‐MSCs will promote tissue regeneration via diverse mechanisms such as immunomodulation, angiogenesis, and anti‐apoptosis [6, 24, 25]. Recently, the administration of MSCs derived factors has become an attractive non‐cell‐based therapy. Hence, comprehensive analysis of these cytokines is an important step in understanding their biological activity and maximizing their preclinical utility. The factors from mBM‐MSCs‐G shared a higher similarity with mBM‐MSCs‐D, raising the possibility that the purer cells were attributable to centrifugation and cell migration. GO analysis of the differentiated secreted proteins indicated the effects of different isolation methods were mainly on immune responses and growth factors. Accordingly, the KEGG enrichment achieved similar results, which involved immune pathways. These results indicated that different isolation methods may have: (a) differing immunomodulatory potency that can regulate the immune microenvironment to provide suitable conditions for regeneration and (b) differing immunogenic inertia, which is involved in immunological rejection. For different diseases, there are specific important factors that are related to disease pathogenesis and progression, and the highly expressed factors (such as MIP‐1b, MIP‐1g, interleukin‐6, progranulin, KC, PF4, periostin, IGFBP‐6, NOV, decorin, TWEAK R, and Pro‐MMP‐9) should be given priority in a certain disease. These findings may help understand how mBM‐MSCs impact neighboring or distant cells with possible consequences for their therapeutic usage. Accordingly, it may provide a framework reference for isolating MSCs from other tissues.

Above all, the bone digestion assay is an optimization for mBM‐MSC isolation for most situations. This method is simple, stable, and practical, being suitable for most experimental needs. However, each separation method has its characteristics and can be chosen based on specific scientific purposes and experimental conditions.

## Conflict of interest

The authors declare no conflict of interest.

## Author contributions

CQ and YL were responsible for study conceptualization. YL, YH, LZ and GS were responsible for methodology. YL, LZ, YH, GS, KW and LB were responsible for formal analysis. YL, YH, LZ, GS, KW and LB were responsible for study investigations. CQ was responsible for resources. CQ was responsible for data curation. YL was responsible for writing the original draft. YL and KW were responsible for reviewing and editing. CQ was responsible for study supervision. CQ was responsible for project administration. CQ was responsible for funding acquisition.

## Supporting information


**Table S1.** Comparison of three mBM‐MSC isolation methods.

**Table S2.** The expression patterns for secretome from the mBM‐MSC groups.

## Data Availability

The data that support the findings of this study are available in the figures and the supplementary material of the published article.

## References

[1] Choe G , Kim SW , Park J , Park J , Kim S , Kim YS , et al. Anti‐oxidant activity reinforced reduced graphene oxide/alginate microgels: mesenchymal stem cell encapsulation and regeneration of infarcted hearts. Biomaterials. 2019;225:119513. 31569016 10.1016/j.biomaterials.2019.119513

[2] Ko HR , Ahn SY , Chang YS , Hwang I , Yun T , Sung DK , et al. Human UCB‐MSCs treatment upon intraventricular hemorrhage contributes to attenuate hippocampal neuron loss and circuit damage through BDNF‐CREB signaling. Stem Cell Res Ther. 2018;9:326. 30463591 10.1186/s13287-018-1052-5 PMC6249960

[3] Maeda T , Sarkislali K , Leonetti C , Kapani N , Dhari Z , Al Haj I , et al. Impact of mesenchymal stromal cell delivery through cardiopulmonary bypass on postnatal neurogenesis. Ann Thorac Surg. 2020;109:1274–81. 31563487 10.1016/j.athoracsur.2019.08.036 PMC7093227

[4] Ezquer FE , Ezquer ME , Parrau DB , Carpio D , Yañez AJ , Conget PA . Systemic administration of multipotent mesenchymal stromal cells reverts hyperglycemia and prevents nephropathy in type 1 diabetic mice. Biol Blood Marrow Transplant. 2008;14:631–40. 18489988 10.1016/j.bbmt.2008.01.006

[5] Dabrowska S , Andrzejewska A , Lukomska B , Janowski M . Neuroinflammation as a target for treatment of stroke using mesenchymal stem cells and extracellular vesicles. J Neuroinflammation. 2019;16:178. 31514749 10.1186/s12974-019-1571-8 PMC6743114

[6] Watson LS , Hamlett ED , Stone TD , Sims‐Robinson C . Neuronally derived extracellular vesicles: an emerging tool for understanding Alzheimer's disease. Mol Neurodegener. 2019;14:22. 31182115 10.1186/s13024-019-0317-5 PMC6558712

[7] Battiwalla M , Hematti P . Mesenchymal stem cells in hematopoietic stem cell transplantation. Cytotherapy. 2009;11:503–15. 19728189 10.1080/14653240903193806 PMC2766085

[8] Dong R , Liu Y , Yang Y , Wang H , Xu Y , Zhang Z . MSC‐derived exosomes‐based therapy for peripheral nerve injury: a novel therapeutic strategy. Biomed Res Int. 2019;2019:6458237. 31531362 10.1155/2019/6458237 PMC6719277

[9] Nakajima D , Watanabe Y , Ohsumi A , Pipkin M , Chen M , Mordant P , et al. Mesenchymal stromal cell therapy during ex vivo lung perfusion ameliorates ischemia‐reperfusion injury in lung transplantation. J Heart Lung Transplant. 2019;38:1214–23. 31474491 10.1016/j.healun.2019.07.006

[10] Wang S , Kim J , Lee C , Oh D , Han J , Kim TJ , et al. Tumor necrosis factor‐inducible gene 6 reprograms hepatic stellate cells into stem‐like cells, which ameliorates liver damage in mouse. Biomaterials. 2019;219:119375. 31374480 10.1016/j.biomaterials.2019.119375

[11] Han Y , Li X , Zhang Y , Han Y , Chang F , Ding J . Mesenchymal stem cells for regenerative medicine. Cell. 2019;8:886. 10.3390/cells8080886 PMC6721852 31412678

[12] Uder C , Brückner S , Winkler S , Tautenhahn HM , Christ B . Mammalian MSC from selected species: features and applications. Cytometry A. 2018;93:32–49. 28906582 10.1002/cyto.a.23239

[13] Shen Z , Li X , Bao X , Wang R . Microglia‐targeted stem cell therapies for Alzheimer disease: a preclinical data review. J Neurosci Res. 2017;95:2420–9. 28643422 10.1002/jnr.24066

[14] Naaldijk Y , Jäger C , Fabian C , Leovsky C , Blüher A , Rudolph L , et al. Effect of systemic transplantation of bone marrow‐derived mesenchymal stem cells on neuropathology markers in APP/PS1 Alzheimer mice. Neuropathol Appl Neurobiol. 2017;43:299–314. 26918424 10.1111/nan.12319

[15] Mosna F , Sensebé L , Krampera M . Human bone marrow and adipose tissue mesenchymal stem cells: a user's guide. Stem Cells Dev. 2010;19:1449–70. 20486777 10.1089/scd.2010.0140

[16] Robinson NB , Krieger K , Khan FM , Huffman W , Chang M , Naik A , et al. The current state of animal models in research: a review. Int J Surg. 2019;72:9–13. 31627013 10.1016/j.ijsu.2019.10.015

[17] Li H , Ghazanfari R , Zacharaki D , Lim HC , Scheding S . Isolation and characterization of primary bone marrow mesenchymal stromal cells. Ann N Y Acad Sci. 2016;1370:109–18. 27270495 10.1111/nyas.13102

[18] Pal B , Das B . In vitro culture of Naïve human bone marrow mesenchymal stem cells: a stemness based approach. Front Cell Dev Biol. 2017;5:69. 28884113 10.3389/fcell.2017.00069 PMC5572382

[19] Méndez‐Ferrer S , Michurina TV , Ferraro F , Mazloom AR , Macarthur BD , Lira SA , et al. Mesenchymal and haematopoietic stem cells form a unique bone marrow niche. Nature. 2010;466:829–34. 20703299 10.1038/nature09262 PMC3146551

[20] Mushahary D , Spittler A , Kasper C , Weber V , Charwat V . Isolation, cultivation, and characterization of human mesenchymal stem cells. Cytometry A. 2018;93:19–31. 29072818 10.1002/cyto.a.23242

[21] Kumar L P , Kandoi S , Misra R , Vijayalakshmi S , Rajagopal K , Verma RS . The mesenchymal stem cell secretome: a new paradigm towards cell‐free therapeutic mode in regenerative medicine. Cytokine Growth Factor Rev. 2019;46:1–9. 30954374 10.1016/j.cytogfr.2019.04.002

[22] Peister A , Mellad JA , Larson BL , Hall BM , Gibson LF , Prockop DJ . Adult stem cells from bone marrow (MSCs) isolated from different strains of inbred mice vary in surface epitopes, rates of proliferation, and differentiation potential. Blood. 2004;103(5):1662–8. 14592819 10.1182/blood-2003-09-3070

[23] Baustian C , Hanley S , Ceredig R . Isolation, selection and culture methods to enhance clonogenicity of mouse bone marrow derived mesenchymal stromal cell precursors. Stem Cell Res Ther. 2015;6(1):151. 26303631 10.1186/s13287-015-0139-5 PMC4549076

[24] Suzuki E , Fujita D , Takahashi M , Oba S , Nishimatsu H . Therapeutic effects of mesenchymal stem cell‐derived exosomes in cardiovascular disease. Adv Exp Med Biol. 2017;998:179–85. 28936740 10.1007/978-981-10-4397-0_12

[25] Wang K , Jiang Z , Webster KA , Chen J , Hu H , Zhou Y , et al. Enhanced Cardioprotection by human endometrium mesenchymal stem cells driven by Exosomal MicroRNA‐21. Stem Cells Transl Med. 2017;6:209–22. 28170197 10.5966/sctm.2015-0386 PMC5442741

