# Supplementary material for: A comparative study of mouse bone marrow mesenchymal stem cells isolated using three easy‐to‐perform approaches
Source: FEBS Open Bio. 2022 Oct 17;12(12):2154–65. doi: 10.1002/2211-5463.13493 (PMC9714364; doi:10.1002/2211-5463.13493)
Supplement: Supplementary file 1 — Table S1. Comparison of three mBM‐MSC isolation methods. [file FEB4-12-2154-s002.docx]

|  | mBMMSCs-A | mBMMSCs-G | mBMMSCs-D |
| --- | --- | --- | --- |
| Age donor | 6-8w adult mice | 6-8w adult mice | 6-8w adult mice |
| Isolation methods | whole bone marrow adherent culture | density-gradient contrifugation method | Bone digestion |
| Isolation mechansim | MSCs have a strong ability to adhere to the Petri dish in low serum medium | the specific gravity of BMMSCs component in the bone marrow | BMMSCs’ migration and adherent growth ability |
| Morphological characteristics | spindle shape  less homogeneous | spindle shape;  more homogeneous | spindle shape;  more homogeneous |
| MSCs antigen markers | good | good | good |
| Differentiation | weak | strongest | Strong |
| Primarily transplantation | Yes | Yes | No |

Supplementary Table 1 Comparison of three mBM-MSCs isolation methods.
