# Supplementary material for: A comparative study of mouse bone marrow mesenchymal stem cells isolated using three easy‐to‐perform approaches
Source: FEBS Open Bio. 2022 Oct 17;12(12):2154–65. doi: 10.1002/2211-5463.13493 (PMC9714364; doi:10.1002/2211-5463.13493)
Supplement: Supplementary file 2 — Table S2. The expression patterns for secretome from the mBM‐MSC groups. [file FEB4-12-2154-s001.docx]

Supplementary Table 2 The expression patterns for secretome from the mBM-MSC groups.

|  | mBMMSCs-A | | | mBMMSCs-G | | | mBMMSCs-D | | | |
| --- | --- | --- | --- | --- | --- | --- | --- | --- | --- | --- |
| AR | 737 | 761 | 942 | 563 | 454 | 425 | | 497 | 492 | 559 |
| Axl | 13,666 | 10,204 | 14,215 | 9,031 | 9,677 | 9,989 | | 12,146 | 10,307 | 9,093 |
| CD27L | 977 | 746 | 837 | 598 | 563 | 770 | | 580 | 466 | 454 |
| CD30 | 683 | 575 | 561 | 441 | 407 | 339 | | 353 | 345 | 372 |
| CD40 | 623 | 658 | 672 | 518 | 552 | 628 | | 375 | 409 | 414 |
| CXCL16 | 35,668 | 31,737 | 34,764 | 26,470 | 31,974 | 30,287 | | 27,285 | 29,397 | 29,012 |
| EGF | 200 | 292 | 285 | 274 | 191 | 255 | | 239 | 311 | 208 |
| E-selectin | 989 | 1,095 | 1,115 | 645 | 524 | 602 | | 1,127 | 1,039 | 1,232 |
| Fractalkine | 870 | 802 | 721 | 2,474 | 2,345 | 2,815 | | 6,742 | 6,712 | 6,908 |
| GITR | 3,275 | 3,763 | 3,380 | 365 | 398 | 281 | | 625 | 456 | 402 |
| HGF | 5,395 | 6,065 | 6,172 | 8,497 | 7,939 | 8,442 | | 10,304 | 12,215 | 10,536 |
| IGFBP-2 | 21,679 | 13,672 | 14,987 | 43,927 | 41,462 | 41,475 | | 32,964 | 35,570 | 47,171 |
| IGFBP-3 | 1,657 | 1,687 | 1,933 | 4,019 | 4,395 | 4,442 | | 5,633 | 5,342 | 6,512 |
| IGFBP-5 | 1,798 | 1,848 | 1,980 | 2,097 | 2,134 | 2,411 | | 1,934 | 1,912 | 1,952 |
| IGFBP-6 | 150,412 | 161,068 | 181,855 | 225,476 | 226,303 | 249,311 | | 247,948 | 234,054 | 262,497 |
| IGF-1 | 80,802 | 96,748 | 93,512 | 83,579 | 88,338 | 94,681 | | 27,639 | 27,622 | 26,039 |
| IL-12p70 | 1,496 | 1,547 | 1,658 | 772 | 758 | 581 | | 664 | 737 | 779 |
| IL-17E | 493 | 446 | 584 | 302 | 303 | 372 | | 387 | 491 | 406 |
| IL-17F | 298 | 335 | 313 | 280 | 212 | 307 | | 211 | 212 | 199 |
| IL-1ra | 12,566 | 19,767 | 11,514 | 35,604 | 29,908 | 31,248 | | 33,274 | 35,699 | 39,035 |
| IL-2 Ra | 329 | 391 | 425 | 270 | 288 | 294 | | 399 | 381 | 399 |
| IL-20 | 528 | 635 | 584 | 401 | 458 | 477 | | 483 | 555 | 421 |
| IL-23 | 1,113 | 1,149 | 1,274 | 722 | 657 | 662 | | 738 | 748 | 752 |
| IL-28 | 544 | 504 | 463 | 463 | 407 | 477 | | 678 | 513 | 701 |
| I-TAC | 1,261 | 1,243 | 1,317 | 696 | 648 | 709 | | 658 | 783 | 840 |
| MDC | 1,467 | 1,724 | 1,631 | 1,952 | 2,491 | 2,620 | | 1,073 | 1,266 | 1,122 |
| MIP-2 | 12,997 | 15,090 | 14,040 | 6,307 | 5,940 | 6,046 | | 2,301 | 2,600 | 2,680 |
| MIP-3a | 2,507 | 2,506 | 2,658 | 4,740 | 4,768 | 4,374 | | 1,466 | 1,456 | 1,477 |
| OPN | 62,541 | 62,803 | 61,248 | 65,980 | 63,003 | 59,209 | | 61,231 | 67,605 | 69,113 |
| OPG | 13,353 | 13,587 | 14,802 | 13,284 | 16,970 | 15,755 | | 14,985 | 15,152 | 17,087 |
| Prolactin | 985 | 1,025 | 844 | 381 | 416 | 452 | | 580 | 469 | 466 |
| Pro-MMP-9 | 45,872 | 49,551 | 50,214 | 21,940 | 21,940 | 22,195 | | 119,995 | 140,316 | 172,160 |
| P-selectin | 101 | 121 | 113 | 202 | 206 | 200 | | 411 | 313 | 424 |
| Resistin | 684 | 795 | 858 | 485 | 459 | 575 | | 374 | 496 | 454 |
| SCF | 714 | 691 | 473 | 520 | 741 | 745 | | 618 | 687 | 694 |
| SDF-1a | 865 | 819 | 855 | 7,066 | 7,513 | 7,255 | | 2,654 | 2,660 | 2,632 |
| TPO | 1,051 | 1,249 | 949 | 854 | 658 | 840 | | 771 | 693 | 697 |
| VCAM-1 | 24,587 | 23,737 | 23,618 | 45,152 | 45,261 | 45,823 | | 52,333 | 50,153 | 52,138 |
| VEGF | 29,407 | 28,109 | 30,163 | 36,077 | 36,554 | 37,277 | | 40,461 | 39,662 | 39,622 |
| VEGF-D | 668 | 544 | 538 | 542 | 576 | 605 | | 531 | 479 | 497 |
| bFGF | 380 | 415 | 420 | 418 | 371 | 408 | | 332 | 235 | 279 |
| BLC | 428 | 409 | 367 | 214 | 202 | 202 | | 281 | 195 | 198 |
| CD30L | 101 | 101 | 96 | 125 | 102 | 108 | | 165 | 166 | 181 |
| Eotaxin | 1,535 | 1,551 | 1,427 | 13,231 | 13,365 | 11,714 | | 62,614 | 63,687 | 66,079 |
| Eotaxin-2 | 594 | 619 | 566 | 665 | 596 | 621 | | 473 | 495 | 499 |
| Fas L | 570 | 477 | 651 | 369 | 449 | 544 | | 468 | 580 | 572 |
| G-CSF | 850 | 881 | 862 | 484 | 513 | 508 | | 423 | 399 | 449 |
| GM-CSF | 898 | 937 | 942 | 1,058 | 1,071 | 1,019 | | 1,088 | 913 | 977 |
| ICAM-1 | 403 | 401 | 409 | 287 | 313 | 301 | | 546 | 428 | 453 |
| IFNg | 1,263 | 1,201 | 1,237 | 1,068 | 1,101 | 1,371 | | 1,092 | 1,337 | 1,247 |
| IL-1a | 497 | 653 | 600 | 532 | 496 | 582 | | 402 | 483 | 388 |
| IL-1b | 817 | 724 | 825 | 733 | 629 | 781 | | 845 | 666 | 953 |
| IL-2 | 1,002 | 1,106 | 1,158 | 895 | 966 | 888 | | 1,105 | 875 | 1,018 |
| IL-3 | 1,149 | 1,167 | 1,457 | 873 | 1,073 | 1,225 | | 1,188 | 1,193 | 1,166 |
| IL-4 | 4,281 | 4,272 | 4,305 | 4,509 | 4,541 | 4,433 | | 4,169 | 4,348 | 4,118 |
| IL-5 | 2,095 | 2,168 | 2,040 | 1,917 | 1,878 | 1,934 | | 1,624 | 1,773 | 1,865 |
| IL-6 | 46,160 | 44,239 | 46,343 | 52,365 | 53,117 | 56,567 | | 105,292 | 137,508 | 122,632 |
| IL-7 | 121 | 140 | 119 | 202 | 201 | 212 | | 87 | 98 | 89 |
| IL-10 | 624 | 606 | 658 | 714 | 720 | 858 | | 712 | 866 | 868 |
| IL-12p40 | 802 | 736 | 972 | 697 | 673 | 658 | | 761 | 743 | 840 |
| IL-13 | 308 | 221 | 297 | 389 | 291 | 360 | | 298 | 371 | 288 |
| IL-15 | 399 | 458 | 384 | 339 | 390 | 320 | | 366 | 397 | 324 |
| IL-17 | 789 | 699 | 633 | 571 | 685 | 522 | | 505 | 609 | 572 |
| IL-21 | 1,851 | 1,697 | 1,820 | 1,787 | 1,554 | 1,715 | | 1,531 | 1,545 | 1,633 |
| KC | 101,118 | 97,538 | 105,240 | 144,081 | 134,200 | 145,115 | | 154,522 | 148,997 | 150,387 |
| Leptin | 128 | 118 | 121 | 101 | 110 | 98 | | 117 | 112 | 116 |
| LIX | 33,457 | 37,112 | 37,255 | 52,304 | 48,252 | 46,974 | | 39,064 | 47,580 | 46,818 |
| MCP-1 | 9,522 | 9,716 | 10,254 | 6,561 | 8,791 | 8,676 | | 10,497 | 11,181 | 12,365 |
| MCP-5 | 3,326 | 4,459 | 4,289 | 17,945 | 19,625 | 20,579 | | 17,373 | 17,153 | 17,486 |
| MCSF | 489 | 555 | 584 | 420 | 445 | 448 | | 599 | 554 | 764 |
| MIG | 979 | 873 | 874 | 846 | 993 | 1,228 | | 839 | 854 | 861 |
| MIP-1a | 1,236 | 1,226 | 1,506 | 2,098 | 1,694 | 1,899 | | 13,195 | 13,340 | 15,468 |
| MIP-1g | 107,861 | 119,913 | 92,429 | 149,402 | 146,809 | 149,043 | | 139,840 | 135,824 | 132,491 |
| PF4 | 95,254 | 92,331 | 100,450 | 150,798 | 151,983 | 132,858 | | 53,667 | 54,677 | 49,771 |
| RANTES | 17,208 | 17,708 | 17,770 | 7,755 | 6,721 | 8,485 | | 12,893 | 13,492 | 15,006 |
| TARC | 759 | 772 | 734 | 1,814 | 2,113 | 2,361 | | 1,167 | 1,195 | 1,200 |
| TCA-3 | 667 | 638 | 602 | 751 | 680 | 687 | | 659 | 519 | 569 |
| TNF RI | 56,132 | 58,737 | 52,527 | 62,486 | 67,539 | 61,704 | | 69,336 | 69,562 | 79,930 |
| TNF RII | 36,176 | 38,739 | 34,836 | 27,188 | 29,321 | 29,633 | | 28,005 | 27,510 | 35,017 |
| TNFa | 981 | 1,094 | 1,128 | 923 | 1,097 | 926 | | 780 | 817 | 952 |
| 4-1BB | 1,116 | 1,221 | 1,235 | 1,143 | 1,119 | 1,165 | | 1,175 | 1,028 | 1,338 |
| ACE | 708 | 934 | 1,051 | 889 | 862 | 731 | | 1,109 | 959 | 853 |
| ALK-1 | 1,044 | 1,004 | 1,057 | 1,036 | 1,042 | 918 | | 1,133 | 1,227 | 1,314 |
| CT-1 | 886 | 814 | 830 | 731 | 883 | 889 | | 724 | 740 | 733 |
| CD27 | 878 | 1,127 | 1,112 | 754 | 478 | 600 | | 961 | 859 | 939 |
| CD40L | 3,281 | 2,987 | 2,919 | 2,422 | 2,555 | 2,571 | | 2,239 | 2,141 | 2,324 |
| CTLA4 | 304 | 364 | 389 | 285 | 291 | 285 | | 299 | 216 | 297 |
| Decorin | 85,877 | 88,485 | 89,917 | 107,521 | 100,138 | 103,660 | | 113,456 | 113,723 | 112,675 |
| Dkk-1 | 364 | 392 | 335 | 284 | 276 | 286 | | 289 | 201 | 199 |
| Dtk | 682 | 604 | 806 | 880 | 832 | 998 | | 978 | 775 | 901 |
| Endoglin | 658 | 456 | 709 | 1,201 | 1,078 | 811 | | 803 | 1,023 | 1,060 |
| Fcg RIIB | 4,008 | 5,165 | 4,336 | 2,737 | 2,447 | 2,343 | | 1,408 | 1,285 | 1,330 |
| Flt-3L | 2,539 | 2,375 | 2,506 | 5,778 | 5,527 | 5,268 | | 6,533 | 6,379 | 6,629 |
| Galectin-1 | 32,154 | 31,899 | 30,861 | 36,788 | 36,269 | 36,961 | | 46,494 | 43,815 | 44,172 |
| Galectin-3 | 33,710 | 39,379 | 33,958 | 32,813 | 31,270 | 31,271 | | 32,962 | 34,185 | 34,300 |
| Gas 1 | 16,186 | 16,450 | 16,843 | 30,557 | 31,187 | 31,538 | | 42,658 | 40,003 | 37,641 |
| Gas 6 | 10,359 | 10,539 | 13,077 | 16,499 | 15,361 | 13,468 | | 6,458 | 8,247 | 8,430 |
| GITR L | 569 | 731 | 858 | 594 | 607 | 505 | | 651 | 658 | 457 |
| HAI-1 | 1,312 | 1,178 | 1,279 | 843 | 676 | 1,039 | | 1,359 | 1,064 | 1,042 |
| HGF R | 1,082 | 1,124 | 1,051 | 1,341 | 1,257 | 1,020 | | 1,162 | 1,017 | 1,035 |
| IL-1 R4 | 783 | 752 | 692 | 855 | 822 | 658 | | 889 | 1,069 | 904 |
| IL-3 Rb | 1,825 | 1,693 | 1,480 | 1,503 | 1,298 | 1,310 | | 1,651 | 1,080 | 1,515 |
| IL-9 | 1,696 | 1,989 | 1,887 | 0 | 0 | 0 | | 0 | 0 | 0 |
| JAM-A | 1,123 | 1,233 | 1,169 | 1,340 | 1,162 | 1,122 | | 1,758 | 1,903 | 2,044 |
| Leptin R | 1,301 | 1,400 | 1,222 | 1,243 | 1,088 | 1,070 | | 1,284 | 1,379 | 1,143 |
| L-Selectin | 391 | 566 | 531 | 858 | 835 | 810 | | 898 | 798 | 829 |
| Lymphotactin | 484 | 506 | 508 | 184 | 178 | 194 | | 105 | 106 | 201 |
| MadCAM-1 | 1,108 | 1,223 | 1,016 | 609 | 858 | 793 | | 889 | 721 | 796 |
| MFG-E8 | 8,925 | 8,251 | 8,701 | 9,597 | 9,455 | 9,443 | | 6,982 | 6,119 | 6,245 |
| MIP-3b | 1,040 | 1,066 | 1,158 | 1,128 | 904 | 1,010 | | 1,451 | 1,498 | 981 |
| Neprilysin | 899 | 894 | 906 | 856 | 846 | 779 | | 792 | 763 | 810 |
| Pentraxin 3 | 20,106 | 19,653 | 19,443 | 21,532 | 21,915 | 21,492 | | 24,274 | 23,562 | 21,408 |
| RAGE | 1,564 | 1,802 | 1,566 | 2,119 | 2,309 | 2,074 | | 1,241 | 996 | 1,200 |
| TACI | 626 | 697 | 571 | 277 | 195 | 285 | | 207 | 199 | 254 |
| TREM-1 | 1,157 | 1,221 | 1,427 | 1,517 | 1,642 | 1,571 | | 1,292 | 1,185 | 1,309 |
| TROY | 982 | 1,028 | 925 | 892 | 978 | 877 | | 985 | 868 | 766 |
| TSLP | 1,438 | 1,437 | 1,463 | 3,575 | 4,105 | 3,912 | | 2,451 | 2,446 | 2,703 |
| TWEAK R | 31,601 | 33,345 | 32,665 | 107,809 | 108,401 | 109,266 | | 85,343 | 83,788 | 88,142 |
| VEGF R1 | 539 | 469 | 387 | 458 | 384 | 396 | | 291 | 304 | 307 |
| VEGF R3 | 1,063 | 1,247 | 1,059 | 1,305 | 1,241 | 903 | | 884 | 705 | 1,006 |
| B7-1 | 453 | 448 | 488 | 1,000 | 1,027 | 1,048 | | 1,224 | 1,207 | 1,481 |
| BAFF R | 162 | 116 | 185 | 102 | 121 | 121 | | 124 | 126 | 123 |
| BTC | 675 | 764 | 706 | 681 | 638 | 708 | | 611 | 623 | 623 |
| C5a | 517 | 611 | 553 | 747 | 763 | 784 | | 883 | 787 | 885 |
| CCL6 | 38,762 | 33,521 | 38,069 | 103,203 | 91,613 | 96,004 | | 80,812 | 61,162 | 80,254 |
| CD48 | 480 | 437 | 445 | 447 | 396 | 469 | | 337 | 392 | 438 |
| CD6 | 125 | 121 | 125 | 114 | 121 | 115 | | 113 | 207 | 205 |
| Chemerin | 2,127 | 2,301 | 1,953 | 1,725 | 1,593 | 1,713 | | 2,035 | 2,071 | 2,280 |
| Clusterin | 381 | 463 | 435 | 376 | 288 | 410 | | 487 | 487 | 489 |
| Lungkine | 702 | 772 | 680 | 635 | 614 | 701 | | 640 | 704 | 699 |
| Cystatin C | 15,448 | 15,059 | 16,615 | 17,324 | 17,405 | 18,623 | | 15,818 | 17,197 | 17,856 |
| DAN | 425 | 478 | 403 | 509 | 411 | 421 | | 554 | 602 | 400 |
| DLL4 | 1,553 | 1,685 | 1,585 | 1,826 | 2,072 | 2,338 | | 1,471 | 1,282 | 1,309 |
| EDAR | 312 | 304 | 304 | 270 | 270 | 286 | | 310 | 276 | 226 |
| Endocan | 406 | 392 | 424 | 283 | 332 | 322 | | 289 | 382 | 297 |
| Fetuin A | 556 | 506 | 490 | 482 | 522 | 594 | | 551 | 548 | 586 |
| H60 | 484 | 475 | 456 | 575 | 549 | 540 | | 477 | 364 | 390 |
| IL-33 | 474 | 411 | 448 | 374 | 364 | 327 | | 289 | 390 | 340 |
| IL-7 Ra | 218 | 299 | 304 | 217 | 183 | 198 | | 269 | 268 | 270 |
| Kremen-1 | 810 | 804 | 700 | 752 | 685 | 656 | | 692 | 749 | 679 |
| Limitin | 626 | 586 | 595 | 375 | 394 | 371 | | 447 | 461 | 448 |
| Lipocalin-2 | 3,953 | 4,231 | 3,332 | 1,107 | 1,059 | 1,336 | | 1,351 | 1,439 | 1,411 |
| LOX-1 | 521 | 601 | 581 | 858 | 745 | 695 | | 524 | 425 | 428 |
| Marapsin | 333 | 311 | 372 | 201 | 211 | 207 | | 272 | 225 | 203 |
| MBL-2 | 297 | 285 | 332 | 266 | 236 | 277 | | 299 | 198 | 292 |
| Meteorin | 826 | 812 | 797 | 1,099 | 1,039 | 1,081 | | 967 | 763 | 726 |
| Nope | 1,110 | 1,140 | 1,166 | 2,018 | 2,138 | 2,063 | | 2,172 | 2,164 | 2,462 |
| NOV | 59,963 | 57,570 | 60,658 | 108,403 | 108,567 | 107,950 | | 97,608 | 93,764 | 96,459 |
| Osteoactivin | 5,772 | 5,258 | 5,439 | 8,496 | 8,917 | 8,922 | | 3,145 | 3,126 | 3,679 |
| OX40 Ligand | 464 | 477 | 453 | 306 | 302 | 307 | | 380 | 488 | 391 |
| P-Cadherin | 309 | 324 | 294 | 394 | 492 | 479 | | 314 | 353 | 380 |
| Periostin | 87,269 | 78,160 | 82,271 | 104,254 | 105,757 | 108,471 | | 101,182 | 106,168 | 112,629 |
| PlGF-2 | 3,538 | 3,726 | 3,870 | 11,931 | 11,536 | 11,069 | | 6,543 | 6,278 | 6,871 |
| Progranulin | 107,277 | 114,721 | 115,650 | 119,606 | 125,899 | 137,915 | | 127,392 | 139,111 | 140,944 |
| Prostasin | 414 | 411 | 396 | 400 | 463 | 379 | | 507 | 456 | 365 |
| Renin 1 | 509 | 566 | 574 | 574 | 583 | 567 | | 1,762 | 1,858 | 2,474 |
| Testican 3 | 409 | 424 | 392 | 260 | 298 | 261 | | 303 | 208 | 328 |
| TIM-1 | 869 | 884 | 820 | 657 | 693 | 696 | | 939 | 887 | 913 |
| TRAIL | 411 | 474 | 585 | 1,132 | 1,159 | 1,146 | | 1,050 | 1,105 | 1,301 |
| Tryptase ┖ | 786 | 730 | 689 | 706 | 717 | 757 | | 593 | 636 | 611 |
| 6Ckine | 539 | 480 | 561 | 367 | 358 | 269 | | 535 | 505 | 393 |
| Activin A | 9,426 | 10,063 | 10,827 | 7,126 | 8,532 | 7,199 | | 4,629 | 4,559 | 5,087 |
| ADAMTS1 | 202 | 199 | 116 | 272 | 239 | 268 | | 282 | 336 | 305 |
| Adiponectin | 757 | 794 | 844 | 720 | 625 | 512 | | 480 | 461 | 392 |
| ANG-3 | 115 | 123 | 98 | 228 | 211 | 205 | | 317 | 314 | 377 |
| ANGPTL3 | 183 | 111 | 112 | 205 | 189 | 202 | | 216 | 272 | 252 |
| Artemin | 107 | 109 | 106 | 10 | 12 | 9 | | 125 | 121 | 114 |
| CCL28 | 226 | 222 | 211 | 125 | 111 | 126 | | 145 | 201 | 198 |
| CD36 | 3,402 | 3,400 | 3,760 | 2,114 | 2,310 | 2,393 | | 2,946 | 2,537 | 3,002 |
| Chordin | 237 | 217 | 202 | 273 | 309 | 294 | | 272 | 271 | 294 |
| CRP | 112 | 115 | 101 | 0 | 0 | 0 | | 126 | 121 | 124 |
| E-Cadherin | 192 | 212 | 181 | 240 | 199 | 238 | | 244 | 218 | 272 |
| Epigen | 213 | 237 | 202 | 268 | 278 | 251 | | 207 | 140 | 214 |
| Epiregulin | 212 | 241 | 219 | 490 | 505 | 638 | | 394 | 409 | 405 |
| Fas | 383 | 314 | 313 | 283 | 300 | 302 | | 317 | 298 | 198 |
| Galectin-7 | 297 | 277 | 292 | 401 | 405 | 392 | | 497 | 389 | 396 |
| gp130 | 145 | 116 | 145 | 301 | 362 | 380 | | 540 | 400 | 517 |
| Granzyme B | 302 | 291 | 364 | 272 | 209 | 294 | | 109 | 171 | 185 |
| Gremlin | 0 | 0 | 0 | 58 | 45 | 45 | | 101 | 112 | 112 |
| IFNg R1 | 334 | 407 | 382 | 269 | 280 | 218 | | 366 | 399 | 376 |
| IL-17B | 91 | 106 | 98 | 154 | 119 | 127 | | 78 | 83 | 89 |
| IL-17B R | 425 | 606 | 558 | 697 | 599 | 715 | | 483 | 608 | 596 |
| IL-22 | 88 | 84 | 94 | 14 | 15 | 15 | | 106 | 93 | 99 |
| MIP-1b | 62,154 | 58,733 | 54,203 | 73,788 | 70,522 | 74,896 | | 104,053 | 90,150 | 91,606 |
| MMP-2 | 21,982 | 23,200 | 21,046 | 37,116 | 37,660 | 38,215 | | 35,939 | 33,884 | 35,629 |
| MMP-3 | 8,139 | 7,724 | 8,240 | 18,334 | 20,754 | 19,547 | | 22,996 | 23,618 | 23,519 |
| MMP-10 | 230 | 201 | 203 | 114 | 108 | 107 | | 364 | 421 | 336 |
| PDGF-AA | 3,305 | 2,153 | 3,130 | 3,805 | 4,056 | 3,795 | | 1,358 | 1,243 | 1,070 |
| Persephin | 176 | 185 | 207 | 198 | 164 | 213 | | 121 | 110 | 159 |
| sFRP-3 | 230 | 245 | 211 | 673 | 590 | 706 | | 306 | 312 | 217 |
| Shh-N | 192 | 207 | 246 | 273 | 286 | 316 | | 274 | 237 | 346 |
| SLAM | 266 | 290 | 289 | 271 | 341 | 280 | | 300 | 290 | 361 |
| TCK-1 | 493 | 502 | 475 | 1,203 | 1,241 | 1,444 | | 1,285 | 1,263 | 1,126 |
| TECK | 213 | 218 | 209 | 110 | 125 | 121 | | 24 | 23 | 24 |
| TGFb1 | 1,200 | 1,236 | 1,228 | 1,347 | 1,941 | 1,855 | | 967 | 1,105 | 1,132 |
| TRANCE | 275 | 252 | 345 | 224 | 230 | 236 | | 360 | 338 | 330 |
| TremL1 | 195 | 195 | 193 | 116 | 127 | 106 | | 169 | 152 | 109 |
| TWEAK | 473 | 389 | 352 | 273 | 283 | 292 | | 464 | 471 | 393 |
| VEGF-B | 312 | 220 | 298 | 309 | 375 | 334 | | 366 | 326 | 352 |
| VEGF R2 | 190 | 126 | 173 | 213 | 203 | 215 | | 183 | 196 | 125 |
